# Sensitivity of GBM cells to cAMP agonist-mediated apoptosis correlates with CD44 expression and agonist resistance with MAPK signaling

**DOI:** 10.1038/cddis.2016.393

**Published:** 2016-12-01

**Authors:** Paul M Daniel, Gulay Filiz, Theo Mantamadiotis

**Affiliations:** 1 Department of Pathology, School of Biomedical Sciences, The University of Melbourne, Parkville, Victoria 3010, Australia

## Abstract

In some cell types, activation of the second messenger cAMP leads to increased expression of proapoptotic Bim and subsequent cell death. We demonstrate that suppression of the cAMP pathway is a common event across many cancers and that pharmacological activation of cAMP in glioblastoma (GBM) cells leads to enhanced BIM expression and apoptosis in specific GBM cell types. We identified the MAPK signaling axis as the determinant of cAMP agonist sensitivity in GBM cells, with high MAPK activity corresponding to cAMP resistance and low activity corresponding to sensitization to cAMP-induced apoptosis. Sensitive cells were efficiently killed by cAMP agonists alone, while targeting both the cAMP and MAPK pathways in resistant GBM cells resulted in efficient apoptosis. We also show that CD44 is differentially expressed in cAMP agonist-sensitive and -resistant cells. We thus propose that CD44 may be a useful biomarker for distinguishing tumors that may be sensitive to cAMP agonists alone or cAMP agonists in combination with other pathway inhibitors. This suggests that using existing chemotherapeutic compounds in combination with existing FDA-approved cAMP agonists may fast track trials toward improved therapies for difficult-to-treat cancers, such as GBM.

Despite the identification of key genetic alterations in glioblastoma (GBM), which drive hyperactivation of key cell signaling pathways regulating cell survival and proliferation, such as the PI3K and mitogen activated protein kinase (MAPK) pathways, therapies targeting pathway factors have not led to improved patient outcome^1, 2^ and postdiagnosis survival for GBM patients is still measured in months. The identification of novel targets in cancers resistant to current therapies, including GBM, is therefore imperative.

One of the key hallmarks characterizing cancer cells is avoidance of apoptosis.^3^ The key factors recognized in the regulation of apoptosis include the antiapoptotic and proapoptotic Bcl-2 family proteins and cysteine protease caspases and are orchestrated by complex receptor and non-receptor triggered events. One underappreciated mechanism that cancer cells use to evade death is via suppression of the 3′5′-cyclic adenosine monophosphate (cAMP) pathway. The phosphodiesterase-4 (PDE4)-selective cAMP inhibitor and antidepressant drug, rolipram, suppresses colon cancer cell migration^4^ and activates apoptosis in chronic lymphocytic leukemia cells.^5^ Rolipram can also induce expression of cyclin-dependent kinase inhibitors, leading to growth inhibition and differentiation of glioma cells.^6^ Importantly, cAMP activation can overcome resistance to classical chemotherapeutics. For example, various colon cancer cell lines, including lines resistant to cytotoxic agents commonly used to treat colorectal cancers, have been shown to be sensitive to specific cAMP activators, which induce growth arrest and apoptosis.^7^ Taken together, existing evidence suggests that modulating intracellular cAMP may affect survival of cancer cells, including cancer cells that are resistant to standard chemotherapeutic drugs.

Despite the promise of cAMP activation as a means to inhibit proliferation and induce apoptosis in cancer cells, the mechanisms involved are not well understood, thereby limiting translation to the clinic. To our knowledge, the only known direct mechanistic link to apoptosis comes from studies on T-lymphoma/leukemia cells first reported by Zhang and Insel.^8^ Indeed, contradictory functions for cAMP have been described in various cell types, including cancer cells, where activation of cAMP in some cells protects cells from cyotoxic drugs, while in other cells cAMP activation promotes apoptosis (reviewed in Insel *et al.*^9^). Recent evidence suggests that tricyclic antidepressant drugs such as imipramine, which elevate cAMP and modulate autophagy may useful in combination with other drugs in glioma therapy as evidenced by improved survival in murine models of GBM.^10^ In addition, differences in cAMP pathway activation have been reported to dictate the susceptibility of cells to malignant transformation and optic tumor initiation.^11, 12, 13^

In the present study, we evaluated the status of the cAMP pathway in several solid cancers using available gene expression data and used a series of human GBM cell lines to identify the therapeutic potential and mechanistic basis underlying the selective response of GBM cancer cells to cAMP agonists, with the view that these mechanisms may operate across many cancer cell types.

## Results

### Suppression of the cAMP pathway is a common feature across different cancers

**Figure 1:**
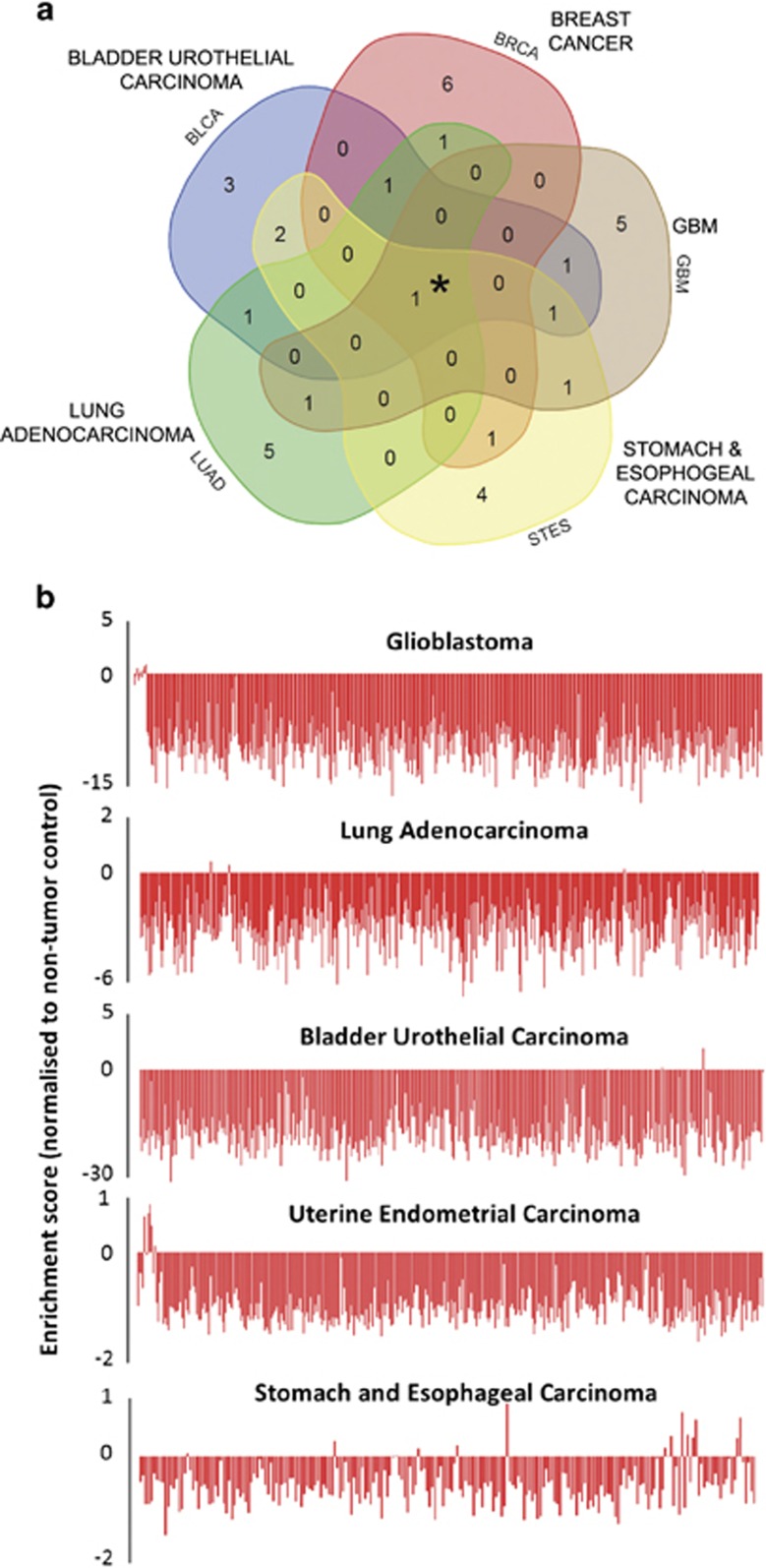
Suppression of the cAMP pathway is a common event in tumorigenesis. (**a**) Five-way Venn diagram displaying overlapping signaling pathways, significantly suppressed in five cancer gene expression data sets, derived from gene set enrichment analysis (GSEA). The top 10 suppressed pathways per cancer indicated were used to generate the Venn diagram (using online software at http://bioinformatics.psb.ugent.be/webtools/Venn/). The analysis shows that one pathway, the cAMP pathway, was suppressed in all cancers, as indicated by the central overlapping region (*). (**b**) Enrichment score of cAMP pathway in individual samples from a set of five TCGA data sets shows that the cAMP pathway is suppressed in almost all patient tumors in the data sets. Scores were normalized to tissue-specific control samples, where zero represents the control score

Intrigued by the observation that decreased cAMP signaling underlies the susceptibility of glial cells to oncogenic transformation by *NF1* heterozygosity,^11, 12^ we utilized gene expression data sets from the The Cancer Genome Atlas (TCGA) to investigate the activation status of the cAMP pathway in several common cancers. Five data sets comprising a total of 2571 cancer samples and 173 tissue-specific non-tumor control samples were analyzed using Gene Set Variation Analysis for pathways differentially expressed between cancer and control samples (Figure 1a). Analysis of glioblastoma, lung adenocarcinoma, bladder urothelial carcinoma and uterine endometrial carcinoma as well as stomach and esophageal carcinoma data sets revealed that all five cancers showed suppression of the cAMP signaling pathway compared with non-tumor controls (Figure 1a). Notably, the cAMP pathway was the only pathway that was consistently enriched in the non-tumor tissues examined.

Analysis of cAMP signaling in individual cancer cases (patient tumors) revealed that suppression of cAMP signaling occurred in 97.84–98.99% across all cancers analyzed (Figure 1b). Bladder carcinoma (average difference 18.9 S.D. from non-tumor) and GBM (average difference of 7.3 S.D. from non-tumor) data sets demonstrated the greatest difference in pathway enrichment compared with non-tumor tissue.

**Figure 2:**
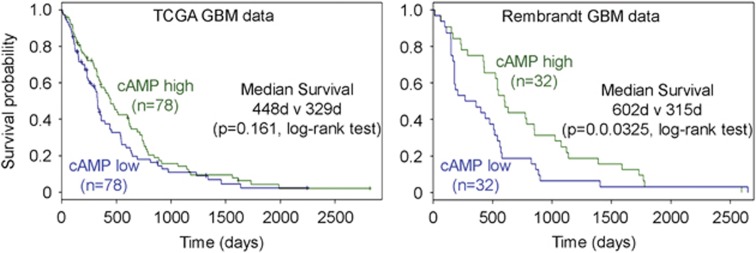
Low cAMP pathway activation signature in GBM correlates with longer survival. Two independent GBM patient cohorts were used. TCGA and REpository for Molecular BRAin Neoplasia DaTa (Rembrandt) gene expression data and corresponding patient survival data was used to generate Kaplan–Meier graphs. TCGA data show non-significant improved median survival as indicated (*P*=0.161) and Rembrandt data show significantly improved survival in cAMP high GBM (*P*=0.0325)

To validate the findings that the cAMP signaling is suppressed in these cancers, we used The Human Protein Atlas^14^ to investigate the expression of protein kinase-A (PKA) catalytic subunit (PRKACA), a key kinase of the cAMP pathway that mediates phosphorylation of multiple downstream cAMP pathway substrates, comparing tumor samples to non-tumor controls (Supplementary Figure S1). In non-tumor tissue, a variable level of expression was seen among the different tissues. Brain cortex and stomach showed the highest PRKACA expression, evidenced by the widespread, intense staining within cytoplasmic regions across the tissue. Bladder and lung showed variable levels of PRKACA expression, while uterine tissue showed weak PRKACA expression. Across all organs examined, tumor tissue exhibited uniformly low expression compared with non-tumor tissue. We did not examine the specific cell types expressing PRKACA across the tissues, but in non-tumor brain cortex, the PRKACA expression was strongest in neurons. Bioinformatic analysis of two independent GBM patient cohort gene expression and survival data shows an association between cAMP pathway activation and survival of GBM patients, where a low cAMP activity expression signature correlates with shorter survival (Figure 2).

### cAMP agonists inhibit growth and trigger apoptosis of GBM cells

**Figure 3:**
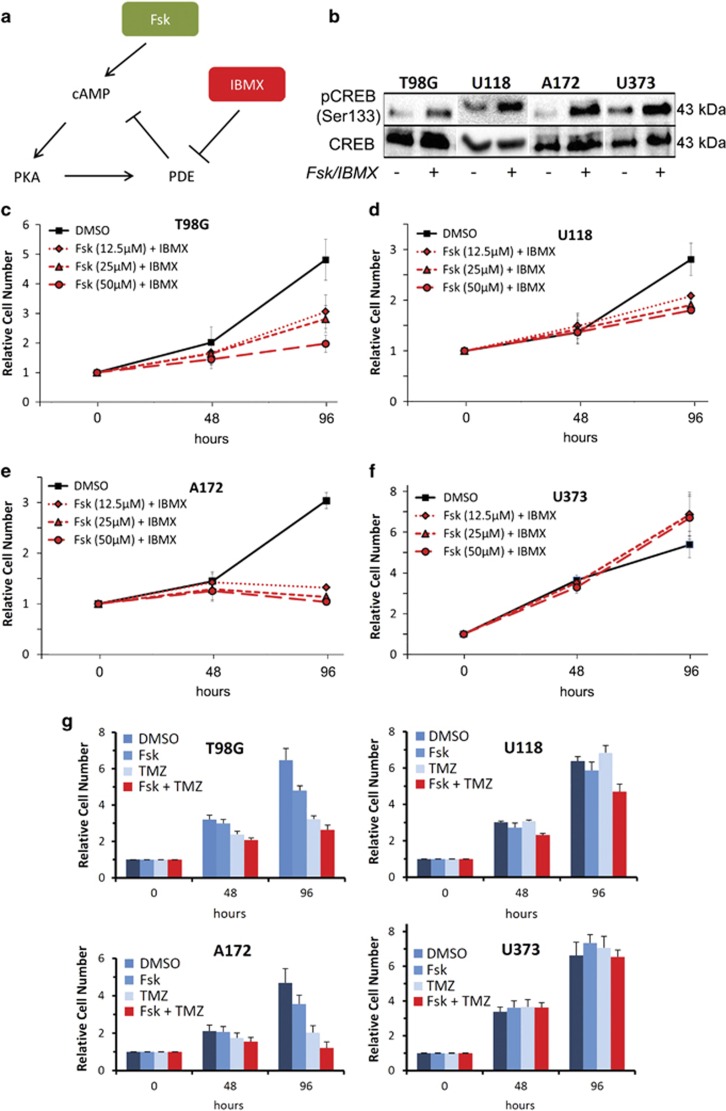
cAMP agonists suppress GBM cell proliferation. (**a**) Schema depicting the molecular targeting of the cAMP pathway and the actions of cAMP-activating compounds Fsk and IBMX, which led to the activation of PKA via cAMP or inhibition of PDEs. (**b**) Western blotting showing the phosphorylation of the PKA substrate, CREB, by Fsk (25 *μ*M) and IBMX (100 *μ*M) in GBM cell lines. (**c**) Dose-independent decrease in cell proliferation/viability by Fsk–IBMX in GBM cell lines T98G and A172 (**e**) but not in U118 (**d**) or U373 (**f**) over 96 h. (**g**) Cells were incubated in the presence of Fsk (25 *μ*M) and IBMX (50 *μ*M), with or without TMZ (200 *μ*M) for 96 h, and the cell number was measured at 48 and 96 h using a Resazurin assay. Results are shown as the ratio of cell number relative to vehicle (DMSO) treatment

Given the recent evidence demonstrating that cAMP agonists can inhibit mouse glioma growth *in vivo*^10^ and the observation that cAMP pathway suppression was a consistent feature across multiple cancers, we investigated the molecular and cellular functions of this pathway in four GBM cells lines, which represent a diverse range of malignant GBM mutational landscapes. To test the response of cells to cAMP activation, forskolin (Fsk), an adenylate cyclase activator, and the PDE inhibitor, 3-isobutyl-1-methylxanthine (IBMX) were used (Figure 3a). As expected, all GBM cell lines examined showed increased phospho-cAMP-response element-binding (CREB) protein (pCREB) expression following exposure to Fsk–IBMX (Figure 3b). Moreover, all cell lines exhibited striking growth differences in response to Fsk–IBMX treatment using a Resazurin-based cell viability assay. Fsk–IBMX inhibited proliferation/viability of T98G cells to the greatest extent (2.3-fold fewer cells; *P*=0.0022; Figure 3c) followed by A172 cells (2.0-fold fewer cells; *P*=2.1 × 10^−5^; Figure 3e) and U118 cells (1.66-fold fewer cells; *P*=0.0019; Figure 3d). U373 cells showed no growth inhibition in response to cAMP stimulation and instead showed a slight increase in cell number after 4 days, compared with control (Figure 3f). All GBM cell lines, including a further two lines tested (LN18 and D270), showed similar effects in response to Fsk–IBMX using an LDH-dependent cell growth/viability assay (Supplementary Figure S2a). Comparing the effect of Fsk–IBMX to various concentrations of the standard GBM chemotherapeutic temozolomide (TMZ) at 96 h exposure to drugs, we observed an equivalent or greater reduction of cell viability by Fsk–IBMX (Supplementary Figure S2b) on T98G, U118 and A172 cells. By contrast, U373 cells were resistant to Fsk–IBMX but sensitive to TMZ, even at the maximum TMZ concentration used (200 *μ*M). Combining Fsk–IBMX and TMZ led to an inhibition of cell growth in three of the four cells lines, with T98G and A172 showing the largest effect, followed by U118. U373 cells did not show a drug-dependent change in growth over 96 h (Figure 3g).

### cAMP agonists induce apoptosis and expression of proapoptotic BIM

**Figure 4:**
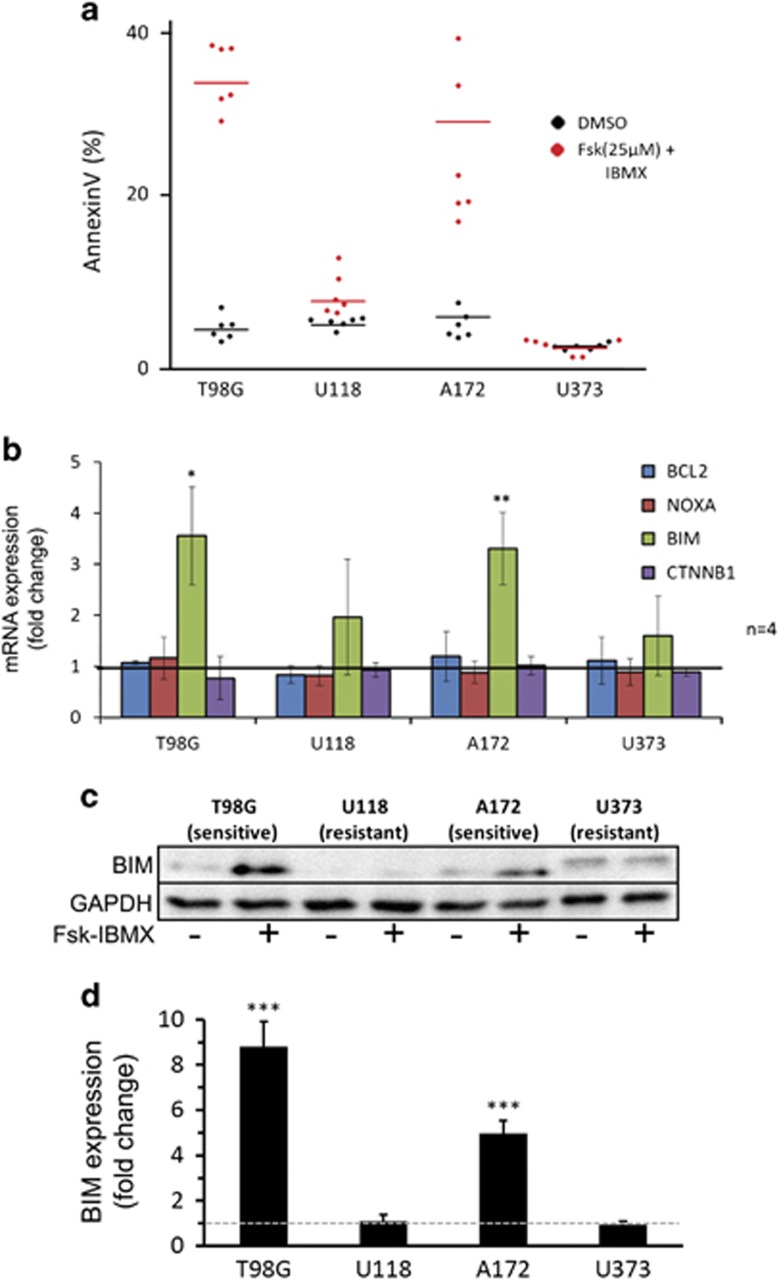
cAMP agonists induce apoptosis in T98G and A172 GBM cells via upregulation of BIM expression but not in U118 and U373 cells. (**a**) AnnexinV FACS quantification comparing vehicle-treated (DMSO) to Fsk–IBMX -treated GBM cells (*n*=3). (**b**) Quantitative reverse transcriptase-PCR analysis of proapoptotic and antiapoptotic genes (*n*=2). (**c**) Western blotting showing BIM expression in cells treated with DMSO or Fsk–IBMX and (**d**) quantification of BIM protein upregulation (*n*=3). Error bars are S.E.M.

A number of studies have demonstrated that cAMP-mediated cell growth inhibition can be accounted for, in part, by enhanced apoptosis.^9, 15, 16^ Using FACS analysis to measure AnnexinV expression, an early marker of apoptosis, we found that 25 *μ*M Fsk–IBMX treatment induced an increase in apoptotic cell number in T98G cells (4.2–23.7% AnnexinV+ *P*=4.52 × 10^−5^) and A172 cells (5.1–18.3% AnnexinV+ *P*=1.20 × 10^−4^) (Figure 4a). By contrast, U118 cells (3.9–6.34% AnnexinV+ *P*=0.33) and U373 cells (2.9–3.1% AnnexinV+ *P*=0.88) did not show significant differences in apoptotic cell number following exposure to Fsk–IBMX, demonstrating a selective response to cAMP-induced cell death among the four GBM cell lines examined (Figure 4a).

We then investigated the ability of Fsk–IBMX to regulate the transcription of several proapoptotic genes. In the two cell lines responsive to Fsk (T98G and A172; Figure 4a), Fsk–IBMX treatment resulted in the upregulation of *BIM* mRNA expression (*P*=0.044 and *P*=0.0013) but did not affect the expression of *NOXA*, *CTNBB1* or *BCL2* (Figure 4b). By contrast, U118 and U373 cells, which exhibited no increased apoptosis in response to Fsk–IBMX treatment, showed no change in BIM expression (Figure 4b). Consistent with the qRT-PCR results, western blotting analysis demonstrated an increase in BIM protein expression in T98G and A172 cells but not in U118 and U373 cells (Figures 4c and d). Together, these results suggest that the cAMP pathway stimulates the expression of the proapoptotic factor *BIM* mRNA and protein but not NOXA, CTNBB1 or BCL2 expression. Thus the upregulation of BIM could contribute to enhanced cell death and the observed reduction in cell number.

### Inhibition of MAPK is necessary for cAMP-mediated BIM upregulation and apoptosis

**Figure 5:**
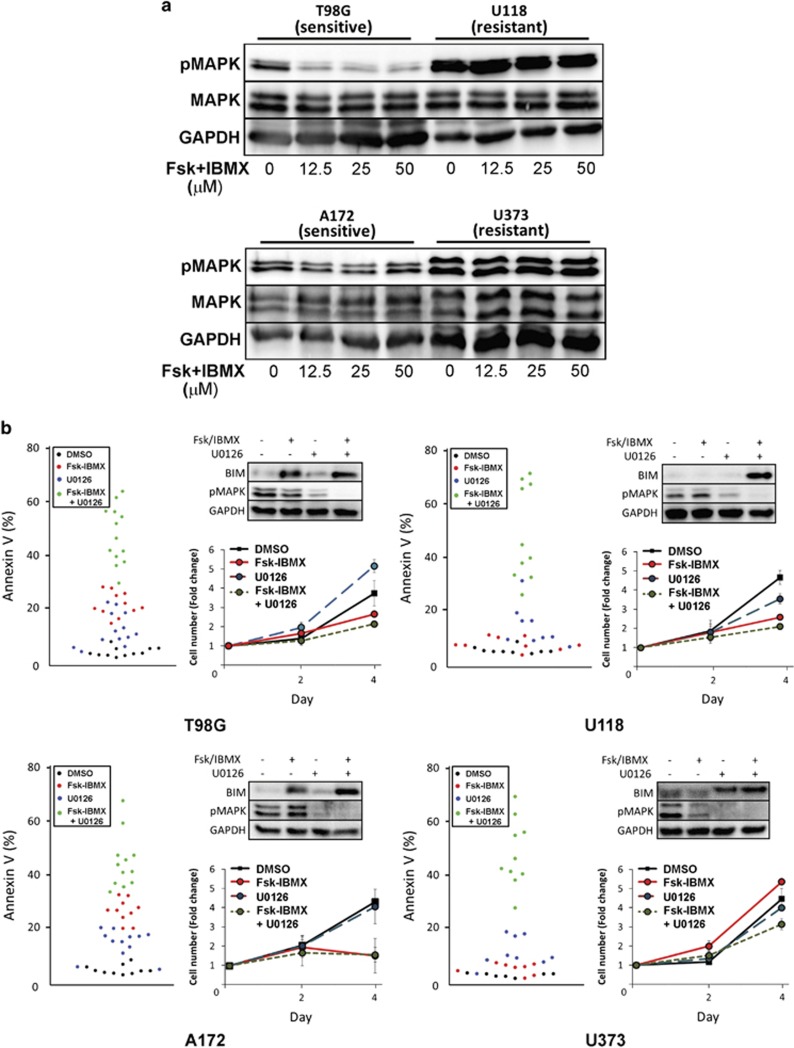
Inhibition of pMAPK is necessary for cAMP-mediated apoptosis. (**a**) Western blotting showing the effect of Fsk–IBMX treatment on pMAPK expression. (**b**) AnnexinV-positive cells showing the extent of apoptosis triggered by combinations of Fsk–IBMX and U0126 in GBM cell lines T98G, U118, A172 and U373 (*n*>4). Top right in each panel shows BIM expression in GBM cells treated with Fsk–IBMX and/or U0126. Lower right graph in each panel shows the effect of Fsk–IBMX and/or U0126 on cell number over 4 days (*n*=2). Error bars are S.E.M.

Having identified a role for BIM in cAMP-mediated apoptosis in T98G and A172 GBM cells, we investigated the mechanism underlying the resistance of U118 and U373 cell lines to cAMP-induced apoptosis (Figure 4a). Examination of the transcriptional regulation of *BIM* showed that the archetypal cAMP-induced transcription factors, CREB and stress-responsive STAT3 do not significantly contribute to Fsk–IBMX mediated apoptosis (Supplementary Figure S3). We further investigated another major cell signaling factor and potent regulator of BIM, the MAPK. Once activated by phosphorylation, MAPK can regulate transcription of *BIM* mRNA via a 3′UTR-mediated mechanism,^17^ as well as regulating the stability of the BIM protein via phosphorylation, which targets the BIM protein for ubiquitin-mediated degradation.^18^ In addition, MAPK activity/phosphorylation can be modulated by the cAMP/PKA pathway.^19, 20^ Phospho-MAPK (pMAPK) expression in all GBM cell lines correlated to sensitivity to Fsk–IBMX (Figure 5 and Supplementary Figure S4), suggesting a key role for MAPK signaling in BIM expression regulation and apoptosis.

To investigate the contribution of MAPK signaling to cAMP-mediated apoptosis, we first determined the effect of Fsk–IBMX treatment on MAPK activity (Figure 5a). In T98G and A172 cells, which we previously determined to be sensitive to Fsk–IBMX, we show that Fsk–IBMX treatment causes inhibition of pMAPK expression. Interestingly, inhibition of pMAPK was not dose dependent, mirroring the dose-independent decrease in cell growth/viability observed in these cell lines (Figures 3c and e). By contrast, U118 and U373 cells, which are insensitive to Fsk–IBMX, did not exhibit a decrease in pMAPK expression, consistent with no change in BIM expression and reduced cell viability and increased AnnexinV expression (Figures 4b and c).

We used the MAPK inhibitor, U0126, to determine the contribution of MAPK to cAMP-mediated apoptosis resistance. As previously observed, treatment of T98G and A172 cells with 25 *μ*M Fsk–IBMX-induced apoptosis. When combined with a low concentration of U0126 (10 *μ*M; Figure 5b), we observed enhanced apoptosis in both T98G (20.19–44.31% *P*=0.0031) and A172 (28.98 *versus* 59.22% *P*=0.0019) cells; a difference reflected by an increase in BIM protein expression. In the cAMP-resistant cells U118 and U373, Fsk–IBMX treatment alone did not increase apoptosis compared with vehicle-treated cells. However, when U0126 was used in combination with Fsk–IBMX, a significant increase in apoptosis was observed in both U118 (Fsk–IBMX 8.78% *versus* Fsk–IBMX–U0126 71.77% *P*=3.37 × 10^−5^) and U373 (Fsk–IBMX 2.25% *versus* Fsk–IBMX–U0126 48.35% *P*=1.92 × 10^−8^) cells. Once again, the difference in apoptosis was mirrored in the change in BIM expression where combined treatment led to an increase in BIM expression from undetectable to detectable. Analysis of each treatment condition on cell number over 4 days revealed a decrease in cells treated with both U0126 and Fsk–IBMX, compared with either treatment alone. These results indicate a key role for MAPK in determining resistance to cAMP-mediated apoptosis.

### RAF isoform dominance determines MAPK-dependent selectivity of cAMP-induced apoptosis

**Figure 6:**
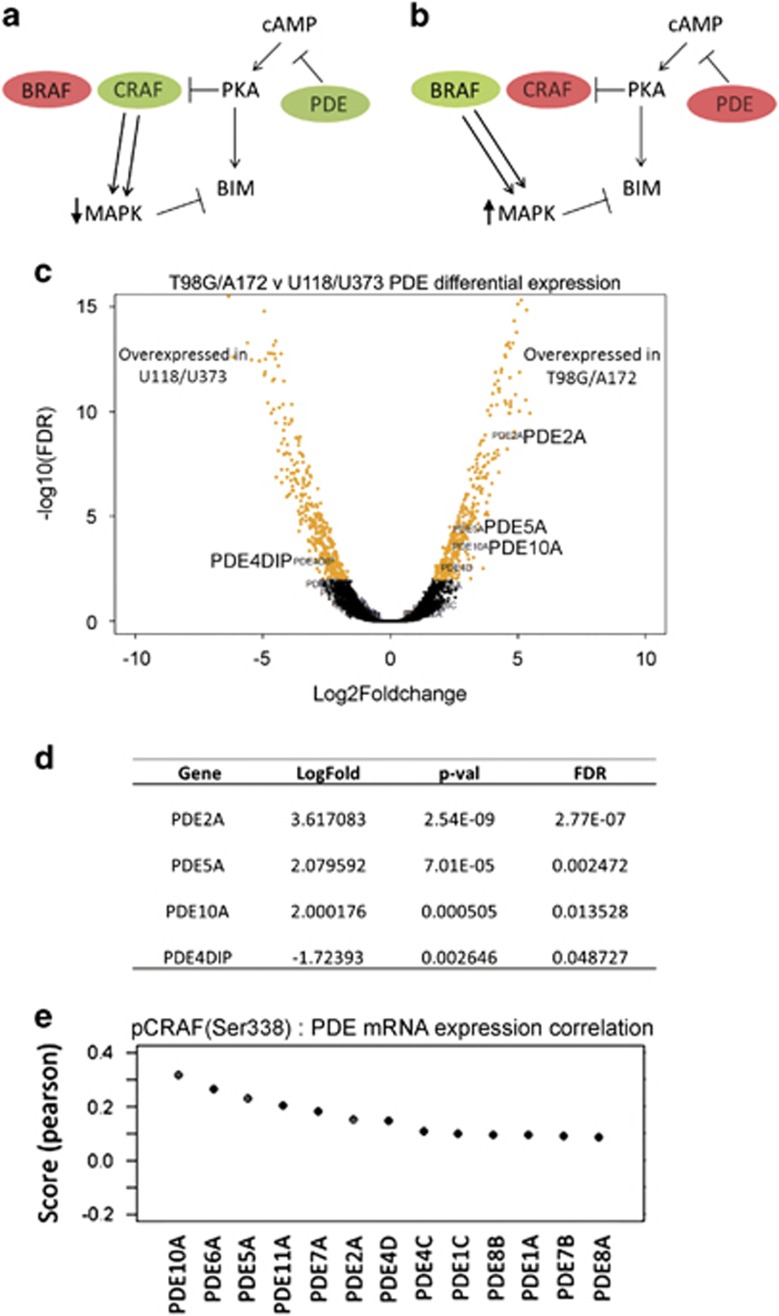
Selective inhibition of pMAPK by cAMP agonists is dependent on RAF isoform dominance. Schema showing the mechanism of RAF isoform-dependent effects in relation to cAMP activation, MAPK pathway activity and BIM expression (modified from Marquette *et al.*^23^). (**a**) In cells where CRAF dominates, cAMP pathway activation can inhibit CRAF, downregulate MAPK activity and increase BIM expression. (**b**) In cells where BRAF dominates, BRAF is unaffected by cAMP signaling, so MAPK activity is high, which in turn inhibits BIM expression. (**c**) Volcano plot generated by gene expression analysis of the relative PDE expression comparing the cAMP-sensitive (T98G, A172) and -resistant (U118, U373) GBM cell lines. (**d**) The four PDEs showing the greatest differential expression between the cAMP-sensitive and -resistant GBM cell lines. (**e**) Co-expression analysis of CRAF phosphorylation and PDE subtype expression in GBM tumors shows that CRAF protein phosphorylation (phosphorylated at Ser338) is high in tumors with high PDE mRNA expression for 13 PDE subtypes examined. CRAF protein phosphorylation data was derived from TCGA reverse phase protein array (RPPA) data sets for GBM and PDE expression from TCGA GBM mRNA expression data sets

The results presented thus far demonstrate a key role for MAPK activity in regulating BIM expression and consequent apoptosis. However, a difference in the ability of Fsk–IBMX to inhibit MAPK activation in GBM cell lines underlies the selective sensitivity to cAMP-induced apoptosis. The cAMP pathway has been reported to differentially regulate RAF isoform dominance, inhibiting CRAF^19, 21^ but activating BRAF,^20, 22^ thereby modulating MAPK signaling. Notably, prior studies have established that RAF isoform dominance in cells is dictated by the expression of PDE factors,^23^ where cells with high expression of PDE, therefore suppressed cAMP-activation, exhibit CRAF isoform dominance, while cells with low PDE expression are BRAF dominant (Figures 6a and b). Therefore, PDE expression level could be used as a surrogate marker for RAF isoform dominance. Importantly, this model provides a rationale for the selective ability of Fsk–IBMX inhibition of MAPK in specific cell lines. We tested this by investigating the expression of PDE family factors in GBM cell lines to determine whether differences in RAF isoform dominance may dictate apoptotic response to cAMP activation.

Using microarray gene expression data from GBM cell lines,^24^ transcriptome analysis of all four GBM cell lines used in our study revealed overexpression of three key PDE family members (PDE2A, PDE5A, PDE10A; Figures 6c and d) in the cAMP agonist-sensitive cells, T98G and A172.

Corroborating the link between PDE overexpression and CRAF dominance, analysis of reverse phase protein lysate microarrays from the TCGA data set revealed that the three PDE family members overexpressed in T98G and A172 cells correlate with CRAF activation (Figure 6e). Taken together, the data presented here suggest that the selective ability of Fsk–IBMX to inhibit MAPK in T98G and A172 cells is due to CRAF isoform dominance, resulting in BIM upregulation and apoptosis. Conversely, Fsk–IBMX is unable to inhibit MAPK in U118 and U373 cell lines, as they are dependent on BRAF, an RAF isoform that is not inhibited by cAMP signaling, therefore insensitive to cAMP agonists.

### CD44 correlates with low pMAPK expression and is a putative biomarker of sensitivity to cAMP-induced apoptosis in GBM

**Figure 7:**
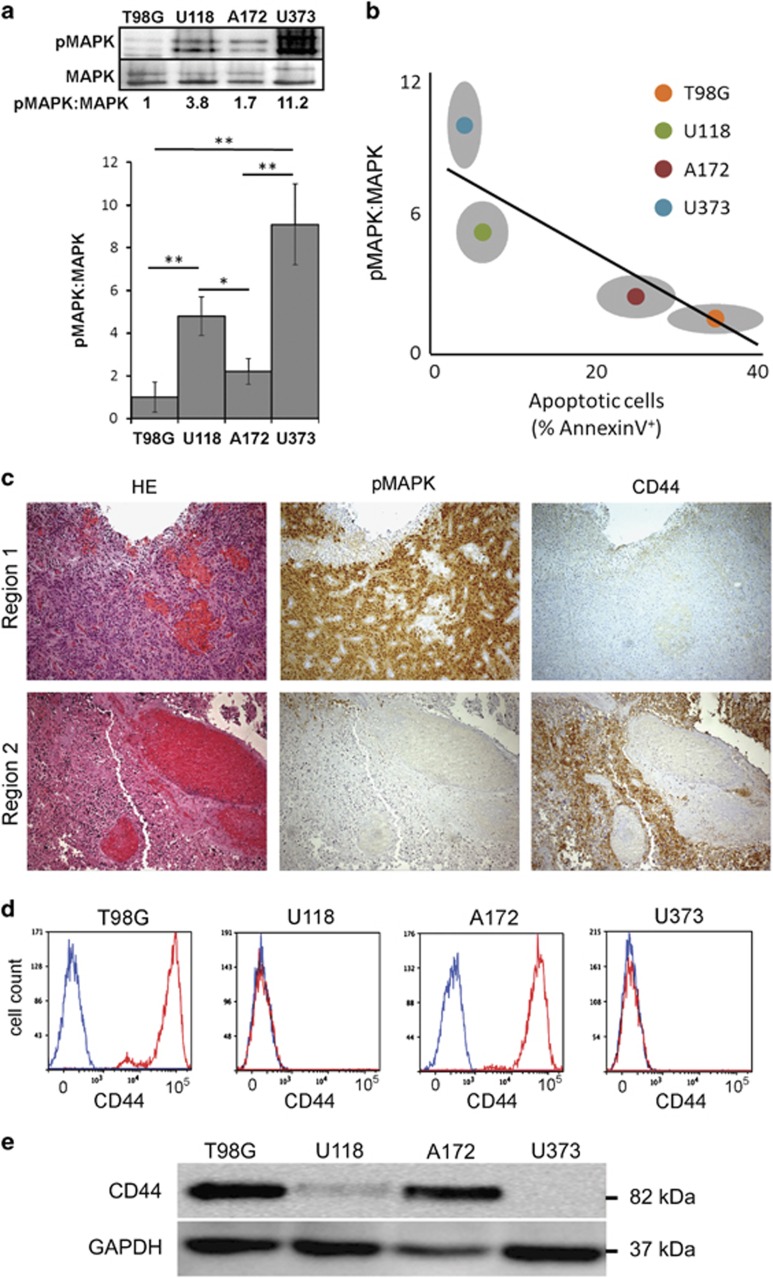
CD44 correlates with low pMAPK expression and is a putative biomarker of sensitivity to cAMP-induced apoptosis in GBM. (**a**) Comparison of pMAPK expression in GBM cell lines (*n*=3). (**b**) Correlation of pMAPK expression and apoptosis upon treatment with Fsk–IBMX (*n*=6). (**c**) Immunohistochemical analysis of a representative GBM specimen showing inverse correlation between pMAPK and CD44. (**d**) FACS analysis of GBM cell lines showing CD44 expression in all sensitive cells and no expression in resistant cells (*n*=3). (**e**) Western blot showing expression of CD44 in the GBM cell lines. Error bars are S.E.M.

A key aspect of cancer treatment when utilizing therapies directed toward a specific pathway or factor is the identification of patients who will likely respond to a specific treatment. Our results establish the MAPK pathway as a key regulator of BIM and key determinant of Fsk–IBMX sensitivity in cancer cells. Furthermore, previous data demonstrate that the kinase activity of the different RAF isoforms varies with BRAF more efficiently activating MAPK, compared with CRAF.^25^ This suggests that cells with lower MAPK activity are dependent on CRAF and are therefore sensitive to Fsk–IBMX treatment. Analysis of MAPK activity in sensitive (T98G and A172) and resistant (U118 and U373) GBM cell lines revealed striking differences in pMAPK expression (Figure 7a). Compared with the most sensitive cell line T98G, the expression of pMAPK was higher in both U118 (4.9-fold greater) and U373 (8.9-fold greater) cells. A172 pMAPK expression was closer (1.9-fold greater) to that observed in T98G cells, indicating an inverse correlation between pMAPK expression and sensitivity to Fsk–IBMX-induced apoptosis (Figure 7b).

In the context of GBM, MAPK is preferentially activated in some subtypes,^26^ suggesting that the use of biomarkers previously implicated in tumor subtype identity may be relevant to the identification of patients with drug-specific sensitive tumors. To this end, we investigated a panel of subtype-relevant markers, including CD44 and Olig2.^27, 28, 29^ Using immunohistochemical analysis, we established that pMAPK expression was heterogeneously expressed within GBM tumors and was inversely associated with CD44 expression (Figure 7c). In tumor regions where pMAPK was highly expressed, CD44 was either absent or expressed at a low level (Figure 7c). By contrast, in regions which expressed low pMAPK, CD44 was highly expressed, suggesting that CD44-expressing cells may be sensitive to Fsk–IBMX treatment. To validate the ability of CD44 in identifying cells that are preferentially sensitive to cAMP reactivation, we analyzed the GBM cell lines for the expression of CD44 (Figure 7d). Expression analysis by FACS and western blottiing confirmed the predictive power of CD44 and cAMP-induced apoptotic sensitivity, as the cell lines which were most sensitive to Fsk–IBMX, T98G and A172 were uniformly CD44 positive. By contrast, U118 and U373 cells, which are resistant to Fsk/IBMX and exhibit higher basal pMAPK expression, were uniformly CD44 negative (Figures 7d and e). To consolidate the inverse association between pMAPK expression, CD44 expression and sensitivity to cAMP agonists, we used two further cell lines with distinct CD44 expression profiles. Comparing GBM cell lines LN18 and D270, we observed that LN18 exhibited low pMAPK, high CD44 expression and sensitivity to Fsk–IBMX treatment, whereas D270 showed the opposite characteristics, consistent with the other GBM cell lines analyzed herein (Supplementary Figure S4).

## Discussion

Identifying novel molecular targets for clinical exploitation is of great significance in cancers such as GBM, where current therapeutic options are limited and patient survival is dismal. Activating tumor-suppressor pathways has been put forward as a means by which tumor growth can be suppressed. However, limitations in targeting specific factors in these pathways restrict their clinical relevance. For example, PTEN is mutated in >60% of primary brain tumors where deletion or inactivating mutations is a critical step for transformation to highest grade of astrocytoma GBM.^30^ Recent studies also demonstrate that PTEN protein can be transferred to PTEN-deficient tumor cells via paracrine mechanism^31, 32^ to inhibit cancer cell growth but the difficulty in re-expressing or delivering PTEN in tumor cells in an *in vivo* setting is a challenge that limits translation to the clinic.

The observation that cAMP is suppressed in cancer compared with non-tumor cells/tissue adds to the evidence demonstrating that the cAMP pathway can act to suppress tumor growth. Interestingly, cAMP pathway activity decreases in an age-dependent manner,^33, 34^ similar to age-associated cancer incidence increase.^35^ There is also evidence that long-term use of medications such as antidepressants that activate the cAMP pathway correlates with a lower risk of certain cancers.^36^ Finally, a causal role for the cAMP pathway and tumor growth attenuation has been established using murine models, directly implicating cAMP signaling in suppressing malignancy.^11^ Together with the data presented herein, there is a growing body of evidence suggesting a role for the cAMP pathway in tumor biology.

Using an integrated approach using bioinformatic and molecular analysis of cancer signaling pathways, we provide the first evidence that the cAMP pathway is suppressed in multiple cancers. We also identify CD44 as a potential biomarker for cell populations that respond best to agonists of the cAMP pathway.

The complexity of response to Fsk–IBMX can be explained by MAPK-driven BIM proapoptotic signaling and provides the rationale for combining cAMP agonists with current treatments targeting key cancer signaling pathways, including components of receptor tyrosine kinase (RTK) pathways that transmit signals via MAPK. This is especially relevant to current clinical practice, as single-agent targeted therapies against components of the RTK pathway have thus far shown limited success in many cancers despite the recognized importance of these pathways in driving tumor growth. For example, 80% of GBM cases harbor either an activating mutation or a copy number increase of the epidermal growth factor receptor (EGFR).^37^ Despite this, clinical trials using small molecules that inhibit EGFR have failed to achieve improved outcomes for GBM patients.^2^ Although drug resistance is a major issue in chemotherapy, another limitation of targeted therapies is likely due to the redundancy of signaling pathways^38^ where inhibition of single factors or pathways has limited effect owing to compensation by other pathways. The data presented in here demonstrate that combining cAMP agonists with inhibitors of MAPK can enhance tumor cell lethality, compared with treatment with either agent alone.

Another property of tumor cells that is receiving increasing attention as a key determinant of therapeutic response is tumor heterogeneity.^31, 39^ This issue highlights the problem where preselection of patients is based on tumor molecular profiling to tailor therapeutic agents to patients. However, this strategy cannot overcome the issue that arises owing to intratumor heterogeneity and the existence of genetically distinct subpopulations of tumor cells. Targeting a mechanism operating more broadly in cancer cells, such as the cAMP pathway, and being able to identify these potentially sensitive cells is an attractive additional therapeutic approach. Addressing this, we identify CD44 as a potential biomarker of cAMP sensitivity. Importantly, we also demonstrate that CD44-negative cells, which are insensitive to cAMP pathway agonists owing to robust MAPK signaling, are rendered sensitive to Fsk–IBMX by concomitant use of MAPK inhibitors. The ability of this treatment to target two mutually exclusive CD44-positive and CD44-negative cell populations, which appear to represent the bulk of tumor cells in GBM, may counteract some of the issues of intratumoral heterogeneity.

The relevance of the results presented here has the potential to be translated to the clinic relatively quickly. Several FDA-approved drugs already exists that target the cAMP pathway and many are commonly used for chronic conditions, including sildenafil (Viagra) and antidepressants, such as rolipram. Particularly relevant to the treatment of brain diseases, antidepressants cross the blood brain barrier thereby bypassing a major pharmaceutical barrier which must be addressed with the development of any new drug which will benefit brain cancer patients. The safety and efficacy of these drugs in treating other conditions suggest that simple combinatorial therapies may be explored relatively easily, as the risks and long-term safety of such drugs are well characterized.

## Materials and methods

### Cell culture

Cells were maintained at 37 °C in a humidified 5% CO_2_ atmosphere. Established human GBM cell lines T98G, U118, A172 and U373 were purchased from ATCC (Manassas, VA, USA) and maintained as previously described.^40^

### cAMP agonist treatment

Previous studies report that the optimal concentrations for robust cAMP activation in different cancer cell lines, including GBM cells, varies between 10 and 50 *μ*M for Fsk and 25–100 *μ*M for IBMX.^7, 41^ Our preliminary experiments showed that using Fsk, an adenylate cyclase activator, or the PDE inhibitor, IBMX, alone (data not shown) was not as efficient at inducing growth inhibition or cell death, compared with using a relatively low-dose combination of the two compounds, between 12.5 and 50 *μ*M for Fsk and 25 *μ*M for IBMX, as indicated for each experiment.

### Cell proliferation/growth assays

Cells were plated onto a 96-well plate and grown under required treatment conditions. On the day of analysis, Resazurin solution (Sigma, St Louis, MO, USA) was diluted in appropriate media and added to wells to obtain a 10% v/v solution before incubation for 3 h at 37 °C. Plates were analyzed using an EnSpire Plate Reader (PerkinElmer, Waltham, MA, USA).

### Flow cytometric analysis

Single-cell suspensions of treated or untreated cells were resuspended in minimal 10% FCS supplemented media before the addition of Muse AnnexinV and Dead cell assay solution (4 : 1 ratio; Merck Millipore, Darmstadt, Germany). The solution was then incubated at room temperature in the dark for at least 30 min before being run using the Muse cell analyzer (Merck Millipore). The population (%) of cells undergoing early apoptosis was identified by plotting AnnexinV expression *versus* PI expression, counting cells which were AnnexinV positive but PI negative.

### Western blotting

Cells were lysed on ice with RIPA buffer. In all, 25 *μ*M of cleared lysate was then run on a 10% Bis/Acrylamide gel volts before being transferred to a PVDF membrane. Membranes were blocked using a 2% milk TBS-T solution for 30 min before probing with primary antibodies diluted at 1:1000 in TBS-T (BIM (Cell Signaling Technology, Danvers, MA, USA), pPKA-C (Cell Signaling Technology), PKA-C (Cell Signaling Technology), GAPDH (Cell Signaling Technology) and phospho-MAPK (Thr202/Tyr204) (D13.14.4E) XP (Cell Signaling Technology).

### Quantitative reverse transcriptase-PCR

RNA was harvested from cells using Trizol (Thermo Fisher Scientific, Waltham, MA, USA) according to the manufacturer's instructions. Reverse transcriptase was carried out using a SensiFAST cDNA Synthesis Kit (Bioline, NSW, Australia). A solution of SYBRGreen and cDNA was mixed with primers specific for BIM (Fwd 5′-AGACAGAGCCACAAGCTTCC, Rev 5′-TCCAATACGCCGCAACTCTT-3′), NOXA (Fwd 5′-GGAGATGCCTGGGAAGAAGG-3′, Rev 5′-CACTCGACTTCCAGCTCTGC-3′), BCL2 (Fwd 5′-GATAACGGAGGCTGGGATGC-3′, Rev 5′-TCACTTGTGGCCCAGATAGG-3′), CTNNB1 (Fwd 5′-GGAGACGGAGGAAGGTCTGA-3′, Rev 5′-CAAATACCCTCAGGGGAACAGG-3′) or GAPDH (Fwd 5′-AGATCCCTCCAAAATCAAGTGG-3′, Rev 5′-GGCAGAGATGATGACCCTTTT-3′) before being run on a Roche LightCycler480. (Roche Diagnostics, NSW, Australia). The average from duplicate samples was used for the ΔΔCt method to calculate fold change in gene expression.

### Immunohistochemistry

Formalin-fixed paraffin-embedded sections were obtained from Associate Professor Kerrie McDonald (UNSW). Human ethics approval for use of GBM specimens used in this study was covered by The University of Melbourne project: 1339751. Slides were first dewaxed and rehydrated by passing the slides through Xylene and then decreasing percentages of ethanol (100, 90 and 70%) before washing under tap water for 2 min. Antigen retrieval was then performed by boiling in citrate buffer for 20 min and left to cool for an additional 20 min followed by a final wash under tap water. Endogenous peroxidase activity was blocked using 3% hydrogen peroxide (Sigma-Aldrich, St Louis, MO, USA) for 10 min at room temperature. Slides were then washed in water and then blocked and permeabilized by incubation in staining buffer (PBS, 2% normal goat serum, 0.5% bovine serum albumin, 0.1% Tween-20) for 30 min. Primary antibody was diluted in fresh staining buffer and then added to the slides and incubated overnight at 4 °C. Antibody concentrations were as follows: 1:4000 phospho-MAPK (Thr202/Tyr204) (D13.14.4E) XP (Cell Signalling), and 1:1000 CD44 (Abcam, UK). The next day, slides were washed with distilled water and incubated with an anti-rabbit or anti-mouse secondary biotinylated antibody (diluted 1:500 in staining buffer) for 30 min at room temperature. During this step, Vector stain ABC solution (Thermo Scientific, Waltham, MA, USA) was made according to the product user manual and then added to sections after a wash step and incubated for 20 min. After washing again in water, slides were incubated with DAB solution (without nickel) for 2–20 min depending on intensity. Slides were then washed under running tap water for 2 min to stop the reaction and then counterstained using hematoxylin and Scott's tap water for a blue nuclear stain. Slides were washed under running tap water for a final time before passing through increasing concentrations of ethanol (70, 90, 100%) and Xylene and then mounting using DPX mounting media (Sigma-Aldrich).

### Gene expression data sets

Analyses performed to generate results shown in this study are based upon original data generated by the TCGA Research Network (http://cancergenome.nih.gov/), Rembrandt^42^ and ENCODE.^43^

### Bioinformatic analysis

All analyses were carried out using R version 3.0.3 (http://rproject.org/). Bioconductor programs (https://www.bioconductor.org/) were used for analyses. Open source GBM TCGA or ENCODE data sets were utilized throughout. Level 3 TCGA data were downloaded on 18 September 2015 from the Broad Institute website (http://gdac.broadinstitute.org/runs/stddata__2015_11_01/data/).

For ENCODE microarray mRNA expression data sets for the GBM cell lines T98G, U118, A172 and U373, data were downloaded from the GEO accession browser (GSE4536).^24^ Data sets were normalized using robust multiarray average, and then genes differentially expressed between T98G/A172 and U118/U373 cell lines were identified with the R package ‘DESeq'.

### Statistical analysis

The statistical program R or Excel (Microsoft, WA, USA) was used for statistical analysis of data. Data are represented as the mean±S.E.M. Differences between groups were compared by unpaired *t*-tests, unless otherwise stated. The following asterisk symbols were used to represent statistical significance: *P<0.05, **P<0.005, ***P<0.0005.
