# Supplementary material for: Sensitivity of GBM cells to cAMP agonist-mediated apoptosis correlates with CD44 expression and agonist resistance with MAPK signaling
Source: Cell Death Dis. 2016 Dec 1;7(12):e2494–. doi: 10.1038/cddis.2016.393 (PMC5261024; doi:10.1038/cddis.2016.393)
Supplement: Supplementary Figures [file cddis2016393x1.pdf]

Fig S1

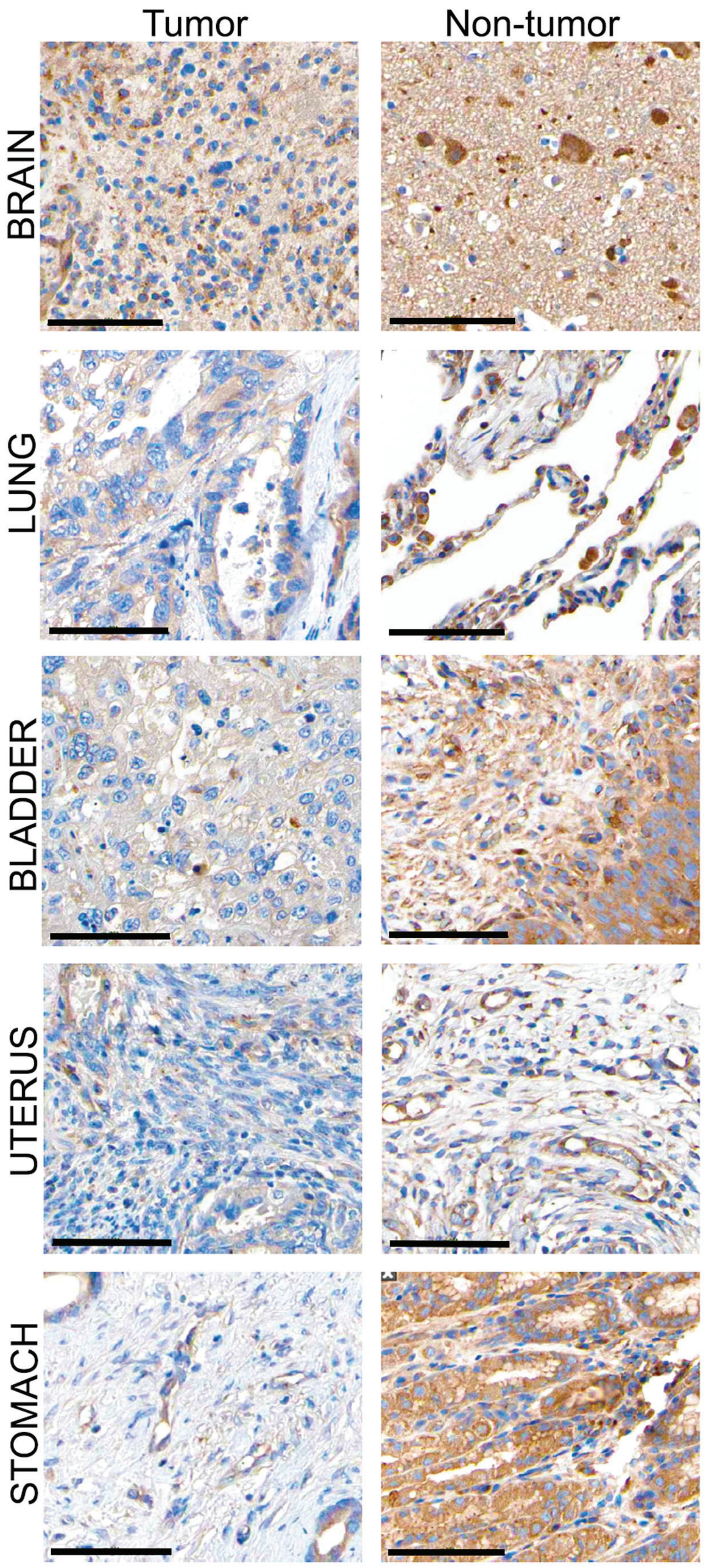

Fig S2

a

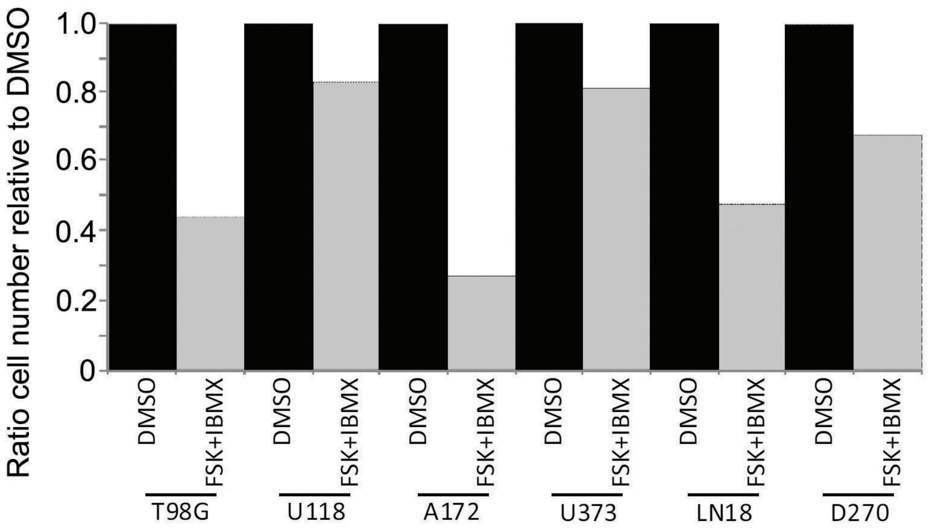
Ratio cell number relative to DMSO
1.0
0.8
0.6
0.4
0.2
0
DMSO
FSK+IBMX
T98G
U118
A172
U373
LN18
D270

b

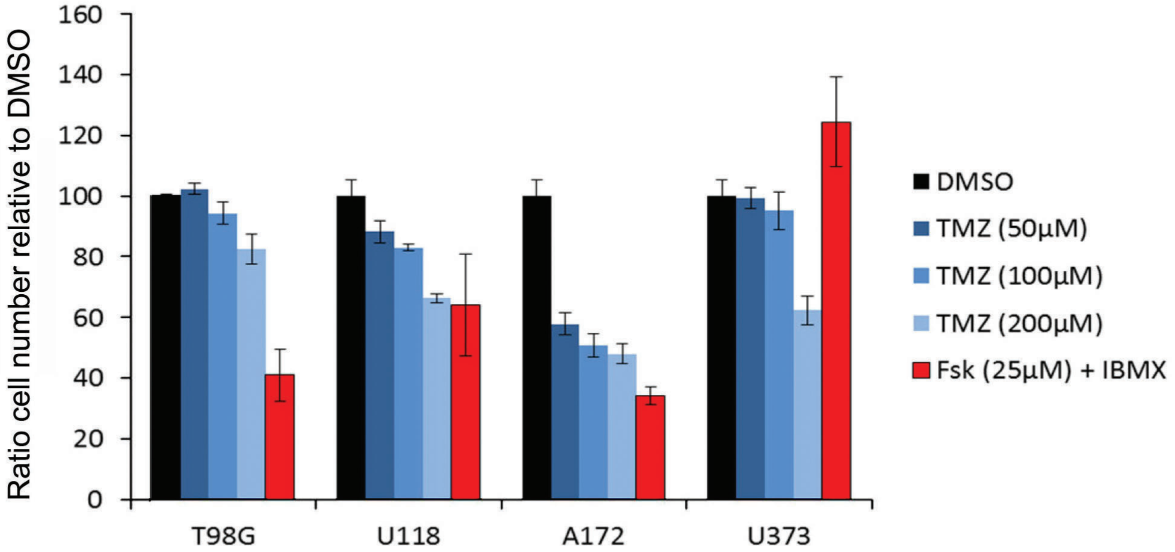
Ratio cell number relative to DMSO
160
140
120
100
80
60
40
20
0
T98G
U118
A172
U373
DMSO
TMZ (50µM)
TMZ (100µM)
TMZ (200µM)
Fsk (25µM) + IBMX

Fig S3

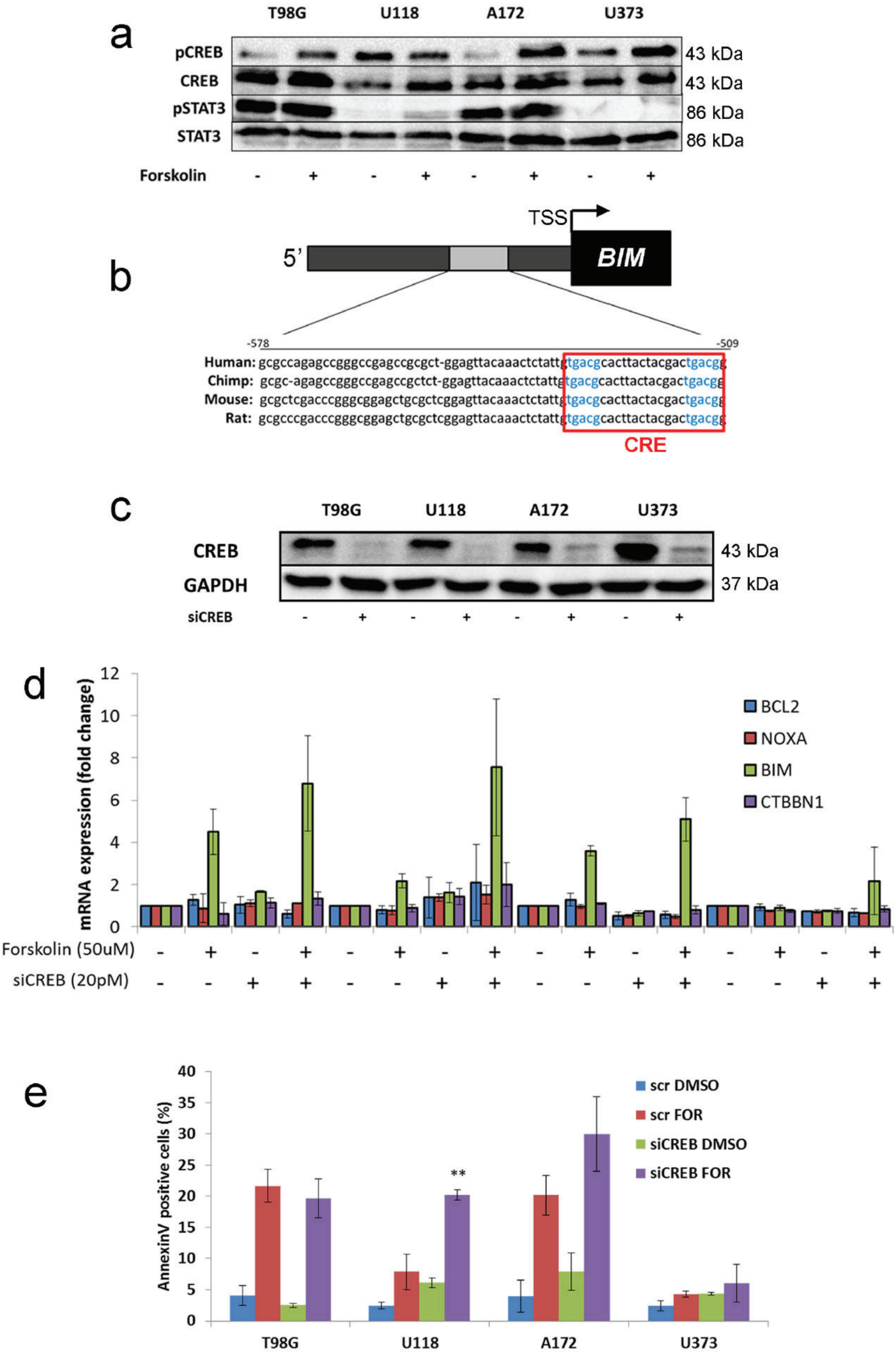

a
T98G
U118
A172
U373
pCREB
CREB
pSTAT3
STAT3
43 kDa
43 kDa
86 kDa
86 kDa
Forskolin
b
TSS
5'
BIM
-578
-509
Human: gcgccagagccgggccgagccgcgct-ggagttacaaactctattgtgacgcacttactacgactgacgg
Chimp: gcgc-agagccgggccgagccgctct-ggagttacaaactctattgtgacgcacttactacgactgacgg
Mouse: gcgctcgacccgggcggagctgcgctcggagttacaaactctattgtgacgcacttactacgactgacgg
Rat: gcgcccgacccgggcggagctgcgctcggagttacaaactctattgtgacgcacttactacgactgacgg
CRE
c
CREB
GAPDH
43 kDa
37 kDa
siCREB
d
mRNA expression (fold change)
BCL2
NOXA
BIM
CTBBN1
Forskolin (50uM)
siCREB (20pM)
e
AnnexinV positive cells (%)
scr DMSO
scr FOR
siCREB DMSO
siCREB FOR
**

Fig S4

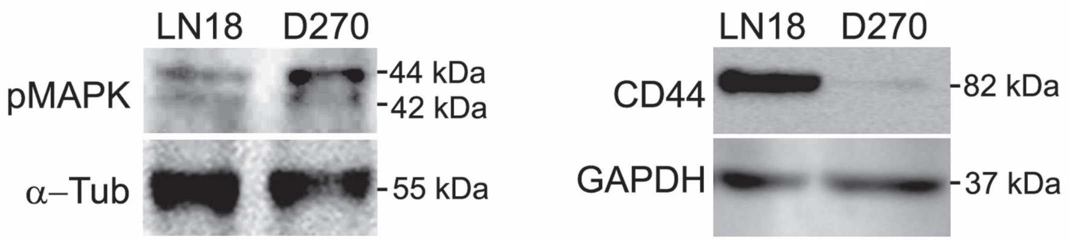

LN18
D270
pMAPK
44 kDa
42 kDa
α−Tub
55 kDa
LN18
D270
CD44
82 kDa
GAPDH
37 kDa

Supplemental Figure 1. The cAMP-dependent protein kinase, PKA, is highly expressed in non-tumor but not in tumor tissue. Expression of the catalytic subunit of PKA, PRKACA, in non-tumor control and tumor tissue. Images are from The Human Protein Atlas (Uhlen et al., 2015).

Supplemental Figure 2. cAMP agonists are as or more effective than temozolomide at inhibiting GBM cell growth. (a) Effect of various TMZ concentrations compared to Fsk (25µM)-IBMX (50µM) treatment on cell number, measured at at 96 hours using an LDH-cell viability assay. Data shown is from one experiment. **(b)** Comparison of Fsk (25µM) and IBMX (50µM) treatment to temozolomide (TMZ) treatment on GBM cell viability at 96 hours following exposure to agonists. Data shown are an average of n=3 independent experiments. Error bars are Standard Error of Mean (S.E.M.).

Supplemental Figure 3. CREB and STAT3 minor contributors to Fsk-IBMX mediated apoptosis. (a) Western blot analysis of change in CREB and STAT3 phosphorylation upon stimulation with Fsk/IBMX. **(b)**Identification of a conserved cAMP Response Element (CRE) site in the *BIM* promoter. **(c)** CREB levels are significantly reduced in siRNA CREB knockdown GBM cell lines. **(d)** qRT-PCR analysis of pro-apoptotic gene expression changes upon treatment of GBM cells with combinations of CREB siRNA and Fsk-IBMX. **(e)** FACS analysis of AnnexinV abundance upon treatment of GBM cells with combinations of CREB siRNA and Fsk-IBMX.

Supplemental Figure 4. Inverse relationship of pMAPK and CD44 expression in LN18 and D270 GBM cells. Western blot analysis of pMAPK and CD44 expression in cells grown in standard 10% FBS supplemented medium.
